# Phase-Sensitive ChEmical Selection (PiSCES) method for fat signal removal in LGE

**DOI:** 10.1186/1532-429X-18-S1-O54

**Published:** 2016-01-27

**Authors:** Martin A Janich, Steven Wolff, Anja C Brau

**Affiliations:** 1 GE Healthcare, Garching, Germany; 2 Advanced Cardiovascular Imaging, New York, NY USA; 3 GE Global Research, Garching, Germany

## Background

Late Gadolinium Enhancement (LGE) allows imaging of infarction by visualizing the accumulation of contrast agent within the myocardium using an inversion recovery (IR) prepared sequence. However, myocardial hyperenhancement can sometimes be poorly detected in the vicinity of epicardial fat because both fat and hyperenhanced tissue appear bright. Thus, a method for fat signal suppression is desired. One previous approach to fat-suppressed LGE was to null fat signal with appropriately timed tip-up/tip-down fat-selective RF pulses [1], but this technique can leave residual fat signal in the image due both to sensitivity to off-resonance and timing requirements to achieve fat nulling. The goal of the present work is to achieve more complete and robust fat suppression by introducing a Phase-Sensitive ChEmical Selection (PiSCES) method that combines phase-sensitive (PS) image reconstruction [2] with customized timing of fat-selective RF pulses.

## Methods

**Figure 1:**
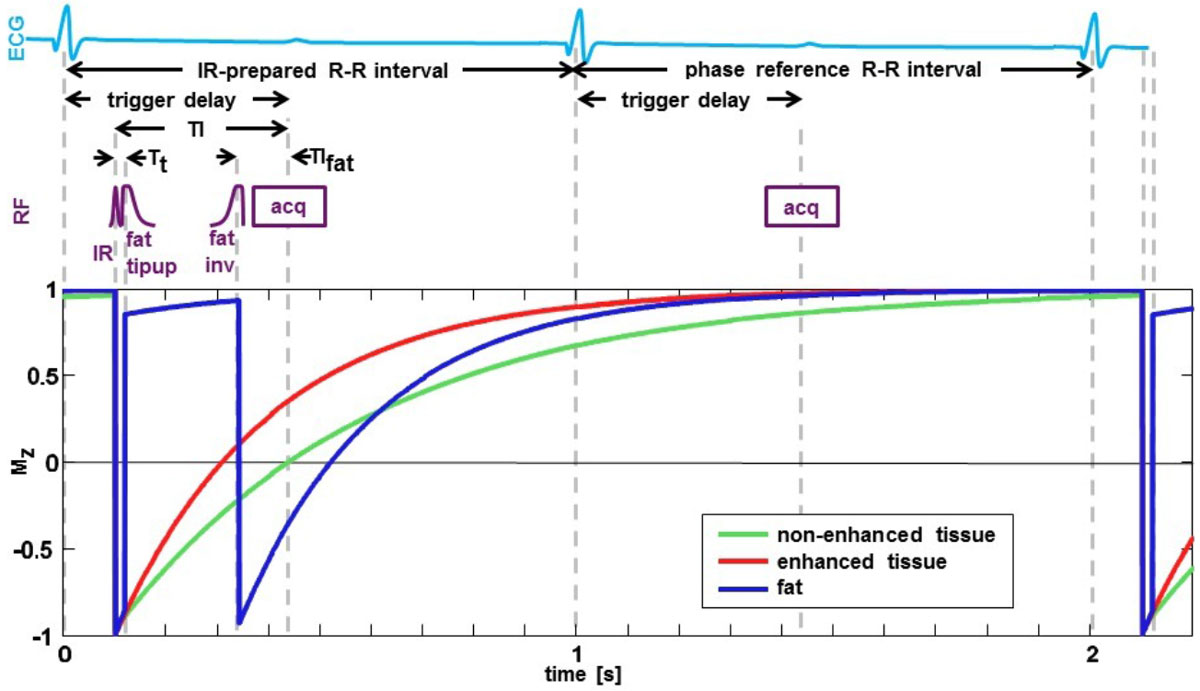
**Pulse sequence timing of PiSCES method, showing inversion recovery, fat tipup, and fat inversion RF pulses, as well as IR-prepared and phase reference data acquisition windows**.

PiSCES is a modification of PS LGE. First, an IR pulse is played with an inversion time (TI) chosen to null normal myocardial tissue. Next, fat-selective tip-up and tip-down pulses are applied, with the tip-down pulse played immediately prior to data acquisition such that fat experiences minimal T_1_ relaxation and longitudinal magnetization of fat signal lies below the transverse plane (M_z_ < 0) during acquisition (Fig. 1).

After PS reconstruction fat appears black due to its opposite signal polarity (M_z_ < 0) vs. hyperenhanced tissue (M_z_ > 0). To achieve this the following condition for TI_fat_, the time between the fat tip-down pulse and the center of k-space acquisition, must be met: TI_fat_ < -T_1,fat_*log(0.5 + exp(-(TI-T_t_)/T_1,fat_) - exp(-TI/T_1,fat_) + 0.5*exp(-2*RR/T_1,fat_)), where T_t_: time between IR and fat tip-up pulses, RR: R-R interval duration.

PiSCES was applied in 8 patients using Discovery MR750w 3.0T (GE Healthcare) and compared to conventional fat-suppressed magnitude LGE with heart rate adaptive timing [3].

## Results

**Figure 2:**
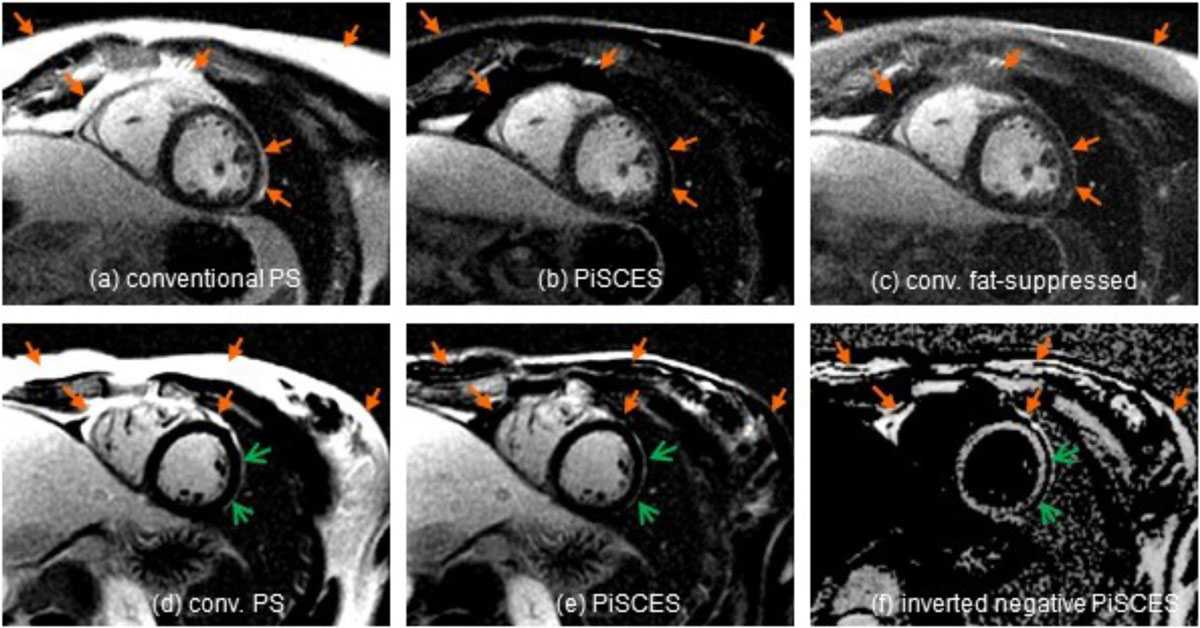
**Exemplary comparison of LGE images in patients without hyperenhancement (a-c) and epicardial hyperenhancement (d-f)**. Filled arrows highlighting fat. Open arrows showing epicardial hyperenhancement.

In 7 out of 8 exams conventional fat nulling resulted in significantly reduced but not completely removed fat signal (Fig. 2(c)), whereas with PiSCES fat signal was completely removed (Fig. 2(b)), as judged by an experienced cardiac radiologist. There was 1 scan with incomplete fat suppression, presumably due to poor shim. In one case pericardial hyperenhancement was better visualized with PiSCES (Fig. 2(e)), confirmed by the negative PiSCES image showing a thin line of pericardial fat (Fig. 2(f)).

## Conclusions

The negative polarity of fat at readout and appropriate windowing of the image leads to complete elimination of fat signal with the new PiSCES approach. Additionally the technique is relatively insensitive to timing of the fat-selective RF pulses: fat appears black as long as fat has negative polarity, thus the method has potential to be more robust across a variety of imaging conditions.
